# Room temperature quantitative liquid concentration device and application to interleukins analysis in a B-cell culture medium

**DOI:** 10.1007/s44211-024-00688-3

**Published:** 2024-11-19

**Authors:** Ruriko Kawanabe, Hidekatsu Tazawa, Kazuma Mawatari, Ayumi Yoshizaki

**Affiliations:** 1 Department of Dermatology, Graduate School of Medicine, The University of Tokyo, 7-3-1 Hongo, Bunkyo-ku, Tokyo, 113-8655 Japan; 2 Graduate School of Information, Production and Systems, Waseda University, 2-7 Hibikino, Wakamatsu-ku, Kitakyushu, Fukuoka 808-0135 Japan

**Keywords:** Concentration, Room temperature, Biological analysis, Medical diagnosis, Interleukin

## Abstract

In biological analysis and medical diagnosis, there is an increasing demand for improving the lower detection limit without deteriorating the quantitativity; however, it is usually challenging. In this study, we utilized a cyclone flow device and established a liquid concentration method. An air cyclone flow induced a liquid cyclone flow in the concentration devices and enhanced the air/liquid interface area, which allowed an effective concentration of liquid from mL to mL at room temperature. The heating temperature and actual temperature inside the liquid were investigated to know the cooling effect of evaporation. The collection efficiency of larger than 98% was confirmed with a standard solution. Finally, the analytical procedure to realize a quantitative concentration was established, and the concentration and quantification of interleukins (IL-8, IL-17, and IL-23) from the supernatant of the B-cell culture medium was demonstrated. The B-cell was stimulated with CD40L, and the supernatant was concentrated 27 times at maximum.

**Graphical abstract:**

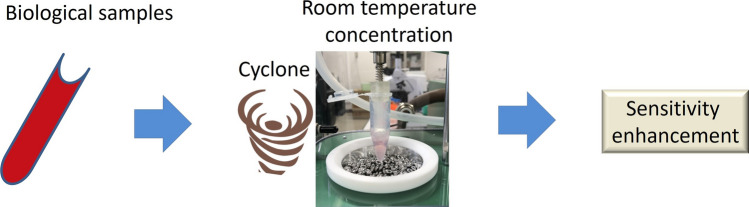

## Introduction

Liquid analysis is frequently utilized in medical diagnosis and biological analysis. The liquid samples widely exist in the environment (water), biological samples (blood, sweat, urine, cell suspension, culture medium, etc.), foods (samples after solid/liquid extractions and water in foods), and chemical synthesis (organic solvents and water). Recently, the requirement for analyzing low-concentration samples has been increasing [1]. Some samples are toxic at very low concentrations [2], which requires highly sensitive analysis. In the medical field, this highly sensitive analysis method will provide new scientific knowledge on the biological functions working at very low concentrations (femtomolar to attomolar). Medical diagnosis also requires high sensitivity to effectively find the symptoms of diseases at an early stage [3]. One of the examples is cytokine. Cytokines are a collective term for low-molecular-weight proteins secreted by cells, and interleukins are an important type of cytokine in the immune system [4]. Interleukins have been implicated in certain diseases and pathologies. For instance, in systemic sclerosis (SSc), an autoimmune disease, interleukins such as IL-6, IL-23, IL-10, and IL-17 secreted by B cells or T cells are known to play crucial roles in the pathogenesis [5, 6]. In atopic dermatitis, IL-13 and IL-4 produced by Th2 cells are known to promote the pathology, and monoclonal antibody preparations targeting IL-13/IL-4 receptors have become common treatments for atopic dermatitis [7]. Thus, sensitive measurement of interleukins is promising for understanding the pathogenesis, diagnosis, and development of cytokine-targeted therapies. However, measuring interleukins at the protein level is often difficult because interleukins secreted by certain cells are at very low concentrations. To solve this problem, we aimed to measure the low levels of cytokines produced by SSc B cells at the protein level.

To realize the highly sensitive analysis, mainly two approaches have been taken. The first approach is to change the detection method. For example, light detection methods were changed from light absorptiometry to fluorometry or chemiluminescence method [3]. If labeling with fluorescent or chemiluminescent molecules is possible, the methodologies can be applied. However, changing the detection method usually accompanies the installation of new and expensive analytical instruments. Therefore, there is a limitation to changing the detection method.

Another approach is to concentrate the liquid samples by decreasing the sample volume. Usually, sample volume can be available at mL order for many analytical situations. However, many analytical instruments require only µL-order sample volume in UV/VIS spectroscopy, Mass spectrometry, high-performance liquid chromatography (HPLC), DNA sequencers, etc. Current microfluidic analysis requires only nL sampling volume [8], and the ultimate small-volume analysis at single cells is being realized utilizing droplet technology [9] and nanofluidic technology [10]. Therefore, there is a gap between the sample volume and detection volume; most of the samples are not utilized and wasted in the analysis. If the mL sample can be concentrated to µL volume, high sensitivity can be achieved. The method does not need new expensive detectors, and basically, there is no limitation for the applications.

There have been reports on liquid concentration methods: mainly, evaporation [11], filtration [12], and extraction [13]. For example, ultrafiltration is applied to macromolecules, where the filter traps the macromolecules on the membrane, and the macromolecules are eluted with a small volume of liquid, which results in concentration. Although ultrafiltration is an excellent method and applied to many macromolecules, the application is limited to large molecules (normally molecular weight larger than several thousands). Due to the filtration procedure, loss of the molecules by adsorption and leakage through the pore affects the quantitative analysis, in particular when the low-concentration samples are treated. Evaporation is a general method to concentrate the samples. Normally, the evaporation process is accompanied by heating to make the evaporation speed higher. However, when considering the biological samples, they usually include proteins inside the volume. Heating denatures the protein and affects the assay results due to the molecular interaction with the proteins. Therefore, the evaporation method at room temperature is desirable. For this, one group reported a cyclone-based concentration method for particle concentration [14]. However, the method has been mainly utilized to dry up the liquid (exchanging the solvents). In biological molecules (not particles), the dry-up process usually leads to the loss of the reaction activity due to the structural change or permanent adsorption of the molecules, which affects the quantitative analysis of the concentration. Therefore, concentration of the biological solutions without drying up the solution is required; however, it was not reported.

In this study, we focus on the mL-level quantitative concentration of biological molecules at room temperature for the analytical application of the cyclone-based concentration method. For this, the temperature inside the concentration device is an important factor for the biological samples. The relationship between the heating temperature and liquid temperature was systematically investigated. Another important factor for the quantitative analysis is the collection efficiency. A standard sample was used to investigate the collection efficiency. Finally, the concentration protocol, including the liquid volume measurement, was established, and the quantitative concentration of interleukins was demonstrated with a supernatant of B-cell culture medium.

## Experimental methods

**Fig. 1:**
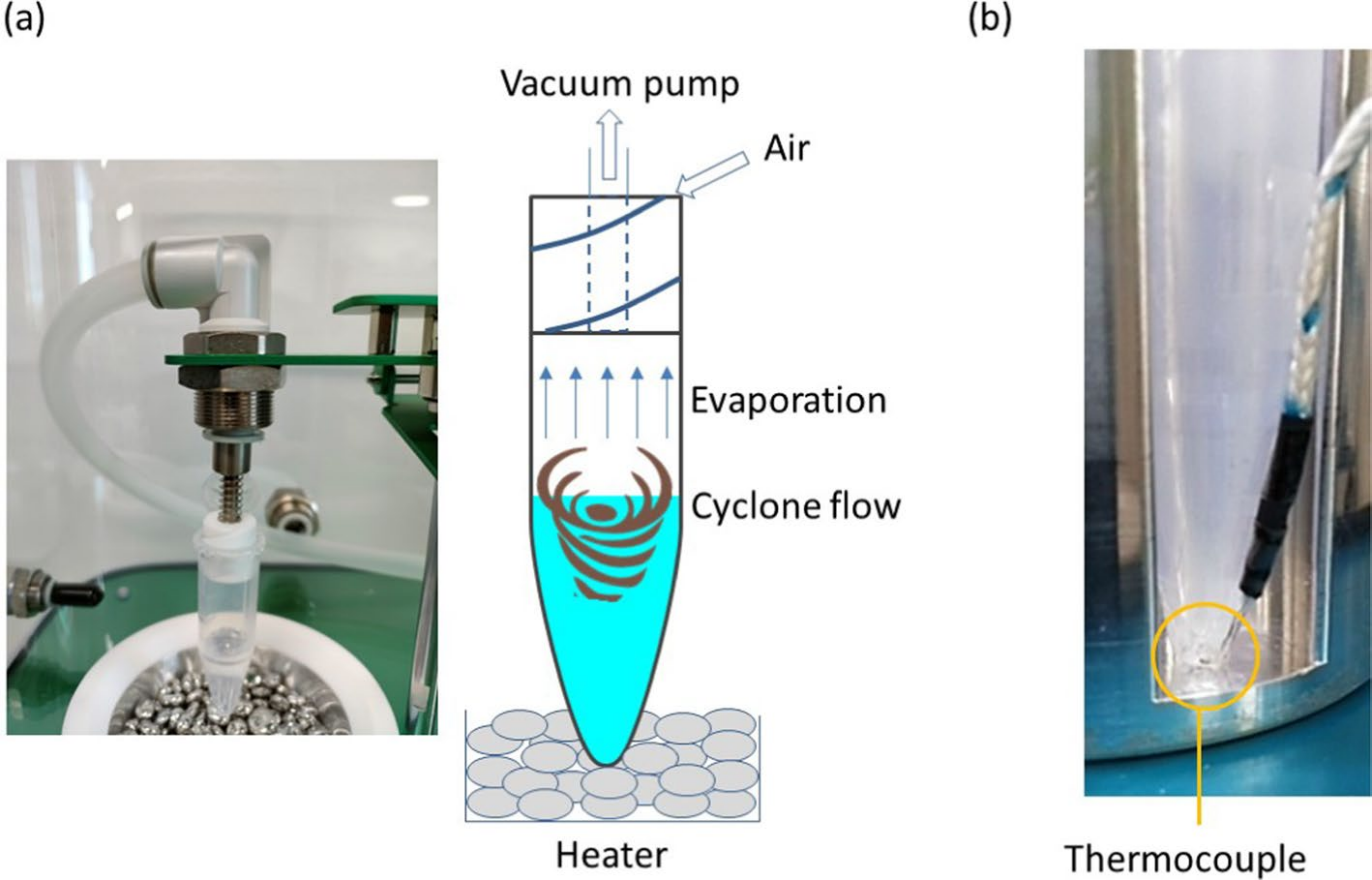
(**a**) Principle of the cyclone-based liquid concentration and (**b**) liquid temperature measurement with a thermocouple

The cyclone-based concentration method was reported elsewhere [14]. Here, the principle is briefly explained using Fig. 1a. The concentration device was capped with a spiral plug made by Teflon, where a spiral channel was formed on the plug. When the negative pressure is applied to air in the concentration device, air outside the device is introduced to inside the device due to the pressure difference. Due to the spiral shape of the channel, cyclone airflow is naturally induced in the concentration device. Then, the shear stress induces a cyclone liquid flow, which enhances the air/liquid interface, and evaporation of the liquid is accelerated even at room temperature. In this experiment, a commercialized system (C1, BioChromato, Inc) was utilized, which equipped a heating unit under the device.

The temperature behavior of the concentrated liquid is important for biological samples. However, there was no systematic study of the effect of evaporation on liquid temperature. For this, the heating temperature was changed with a heater, and the liquid temperature was measured at the bottom of the device using a thermocouple (TM-902, Smgda-JP) with a diameter of 1 mm. A small hole (appx. 1.1 mm) was formed on the wall of the device, and the thermocouple was injected inside to measure the liquid temperature as shown in Fig. 1b. The injection length is 1.5 mm. An adhesive was utilized to fill the gap between the thermocouple and the device.

The collection efficiency after concentration was evaluated using an aqueous solution containing 40 ppm of glycyrrhizic acid dipotassium salt (FUJIFILM Wako Pure Chemical Co). A 1.5 mL tube was utilized to concentrate the 0.1 mL solution and a 5 mL tube was used for the 1 mL solution. The collection efficiency was determined by the concentration of glycyrrhizic acid when water was added after the evaporation process to be equal amount before the evaporation process. The concentration of glycyrrhizic acid was determined by UV absorbance at 258 nm.

**Fig. 2:**
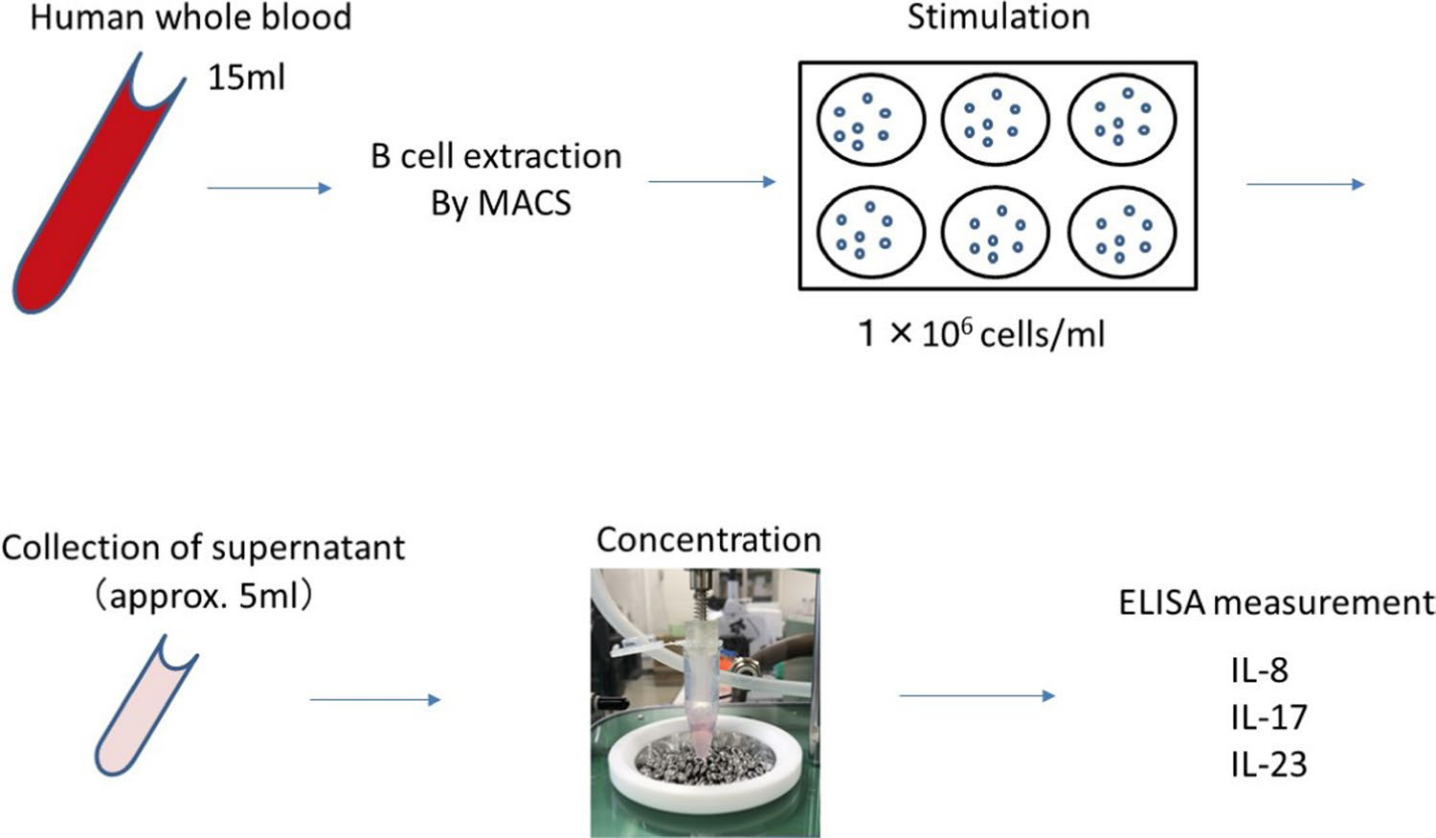
Protocol for determining the concentration of interleukins in a B-cell culture medium

As an application, the concentration device was applied to interleukins analysis. The protocol is shown in Fig. 2 B cells were extracted from peripheral blood of patients using MagCellect Cell Selection Kits & Reagents (MACS, R&D Systems). The extracted B cells were resuspended in RPMI medium containing 10% fetal bovine serum, antibiotics, antifungal agents, and glutamine at a concentration of 1 × 10^6^ cells/ml, and cultured for 48 h with CD40 L (1 µg/ml), followed by a 5-h incubation with PMA (50 ng/ml, Sigma), ionomycin (500 ng/ml, Sigma), and monensin (2 μM, eBioscience). The culture supernatants were promptly stored at − 20 °C after collection. Prior to concentration, the samples were thawed naturally, and the mass of the culture medium before concentration was measured using an electronic scale to calculate the volume. After concentration, the mass of the culture medium was measured using the electronic scale, and the concentration ratio was determined from the ratio of the mass of the culture medium before and after concentration. All operations, except for the concentration process, were performed on ice. The concentrations of IL-8, IL-17, and IL-23 in the B cell culture supernatants were measured using ELISA (enzyme-linked immunosorbent assay) kits: Human IL-8 ELISA (R&D Systems, D8000C), Human IL-17 ELISA (R&D Systems, D1700), and Human IL-23 ELISA (R&D Systems, D2300B). IL-17 was investigated twice. For the first IL-17 measurement, the concentrated samples were diluted with assay diluent to achieve dilutions of 7.1-fold and 2.3-fold before conducting the assay.

## Results and discussion

**Fig. 3:**
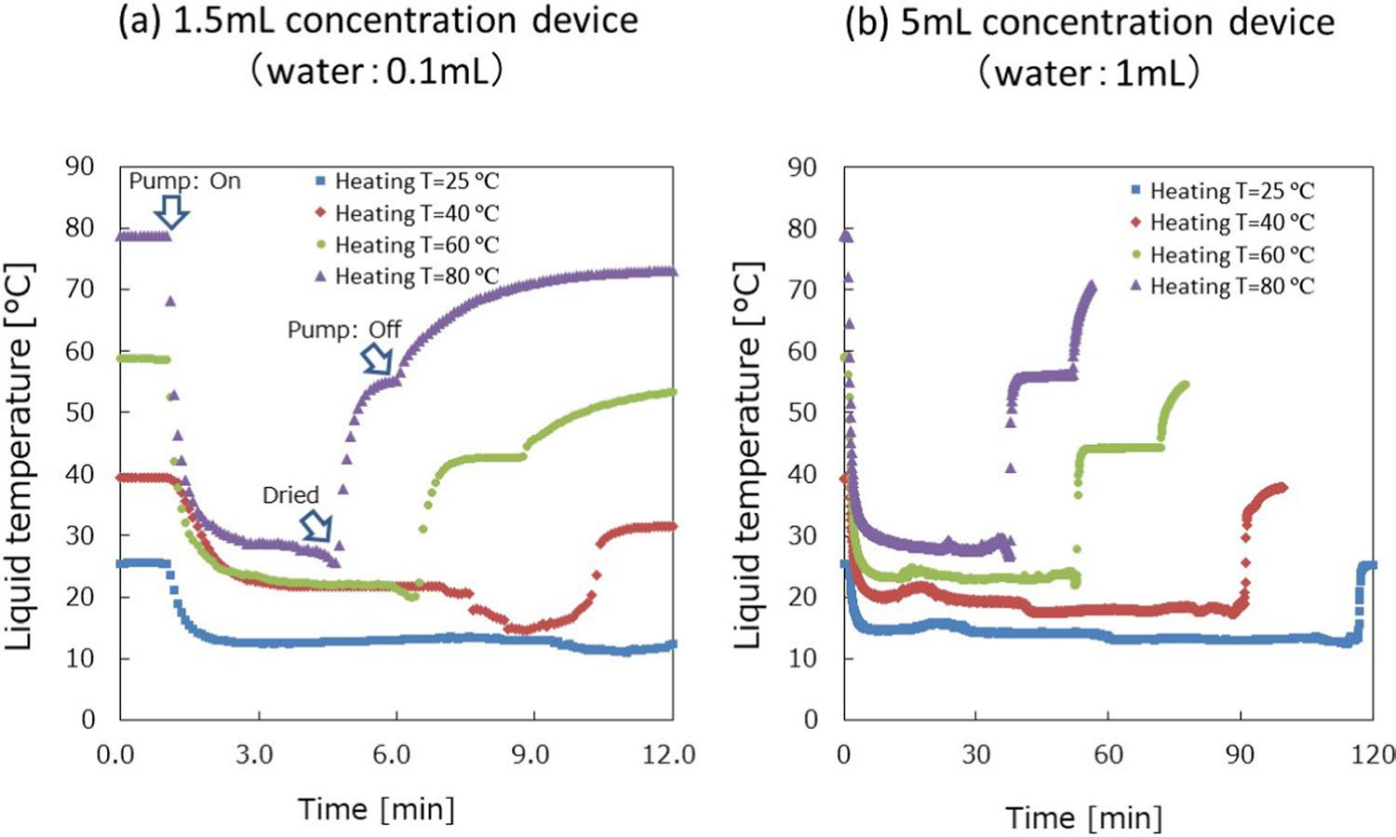
Results of liquid temperature measurements: (**a**) 1.5 mL concentration device for 0.1 mL water and (**b**) 5 mL concentration device for 1 mL water

The temperature profiles of liquid for different heating temperatures are shown in Fig. 3. After turning on the vacuum pump, cyclone flow quickly started in less than 1 s, and the temperature decreased due to the evaporation. During the evaporation, the temperature gradually decreased and quickly increased after the liquid dried completely. The result also shows that the liquid temperature is below 37 °C even for the heating temperature of 80 °C. This means that high temperatures can be applied for the concentration if the heating starts one minute after turning on the vacuum pump. The time to decrease the temperature from 80 to 37 °C is less than 1 min from the result. That means the gradient temperature profile from 37 to 80 °C in 1 min will secure the liquid temperature less than 37 °C for biological samples. For 1 mL samples, approximately 30 min was needed to dry up the solution at 80 °C, while 115 min was needed at room temperature. The dry-up time depended on the humidity of air introduced to the concentration device. Based on this result, depending on the timing of the heating, 80 °C can also be utilized to accelerate the concentration and reduce the time.

Collection efficiency was evaluated for 1.5 mL and 5.0 mL concentration devices as shown in Table 1. The liquid volume and pumping flow rate were both changed. From the result, the collection efficiency significantly decreased for large sample volumes and high flow rates, when the 1.5 mL device was used. This is due to the liquid height of the cyclone flow becoming higher, and some liquid (sometimes forming droplets during the cyclone flow) is sucked into the vacuum pump, which is a loss of analytes and decreases the collection efficiency. In the case of the 5 mL tube, the liquid height was low enough to avoid the droplet sucking, and high collection efficiency (> 98%) was achieved. In this way, the concentration device can provide the quantitative concentration platform for analytical technologies by considering the appropriate volume (concentration device and liquid) and the flow rate of the vacuum pump.

**Table 1:** Results of collection efficiency (%) measurements for different water volumes and vacuum pump flow rates (*n* = 8 for each condition)

Liquid volume [mL]	Vacuum pump [L/min]			
	2	3	4	5
(a) 1.5 mL concentration device				
0.1	–	–	96 ± 3.3	96 ± 0.9
0.2	–	99 ± 1.4	97 ± 1.0	90 ± 5.0
0.3	99 ± 0.4	98 ± 2.2	90 ± 3.1	84 ± 5.5
0.4	100 ± 0.3	97 ± 4.3	83 ± 4.3	–
0.5	100 ± 0.3	91 ± 5.6	–	–

Liquid volume [mL]	Vacuum pump [L/min]			
	2	3	4	5
(b) 5 mL concentration device.				
0.5	–	–	99 ± 0.5	99 ± 1.0
1	–	–	100 ± 0.3	99 ± 0.6
1.5	–	–	99 ± 0.6	99 ± 1.5
2	–	–	99 ± 0.3	98 ± 1.2
2.5	–	–	99 ± 0.7	98 ± 1.2

**Fig. 4:**
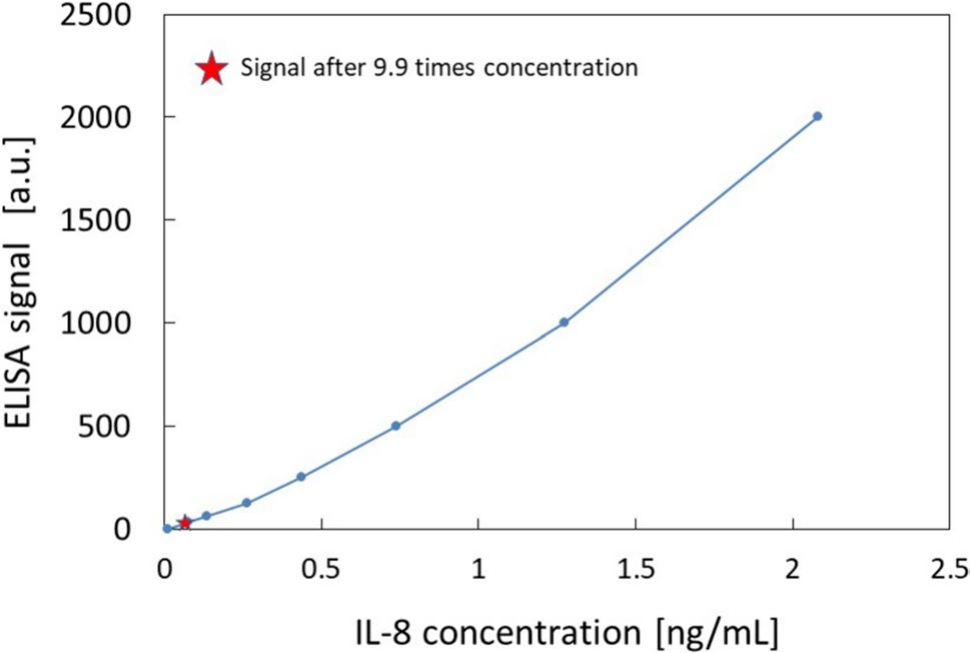
A calibration curve of IL-8 by ELISA and measurement of IL-8 in the concentrated B-cell culture medium

Finally, the concentration device was applied to the quantitative concentration of interleukins at room temperature. The results of ELISA before and after concentration are shown in Fig. 4. The IL-8 levels in the culture supernatant were below the detection limit before concentration (measurement range 31.2–2000 pg/ml), while the 9.9-fold concentrated sample could be measured at 70 pg/ml (Fig. 4), which means that the concentration of the original solution was 7.1 pg/mL. A previous study measuring IL-8 in B cell culture supernatants of various cell lines of healthy human and lymphoma origin has reported that IL-8 levels ranged from 5 to 800 pg/ml in healthy human B cells and 0–3200 pg/ml in lymphoma B cells [15]. There are no reports examining IL-8 levels in SSc B cells, but the result of 7.1 pg/ml seems to be acceptable. The analytical accuracy is mainly determined by ELISA assay, which is CV = 8% at maximum based on the ELISA-kit manufacturer’s data. Although IL-17 (measurement range 31.2–2000 pg/ml) was concentrated up to 26.7-fold and IL-23 (measurement range 39.0–2500 pg/ml) was concentrated up to 15.8-fold, both concentrations were below the detection limit. A previous study measuring IL-17 levels in culture supernatants after B cell stimulation has shown that without other cytokine stimulation, IL-17 concentrations in B cell culture supernatants are below 5 pg/ml (limit of detection), indicating that IL-17 concentrations in B cell culture supernatants are very low without specific stimulation [16]. Therefore, further concentration may have been needed to detect IL-17. For IL-23, there are reports showing gene expression of IL-23 in B cells [5, 17], but there are no reports showing expression of IL-23 at the protein level in B cell supernatant. Therefore, the concentration may be also very low, and further concentration may allow the detection of IL-23 by ELISA. Based on the results, the effectiveness of concentration at mL level was demonstrated in the detection of IL-8 and showed the requirement of further concentration for IL-17 and IL23.

## Conclusion

The quantitative concentration method was established utilizing a cyclone-based concentration device. Temperature behaviors of an aqueous solution for different heating temperatures were investigated, and the temperature decrease by evaporation was quantitatively shown. The collection efficiency was more than 98%, which was good for quantitative analysis. Finally, the method was applied to real biological samples using B-cells, and the concentration effect was confirmed. This is the first demonstration of quantitative concentration of biological molecules at room temperature, and the method will be widely applied to bioanalysis and medical diagnosis.

## Data Availability

All the relevant data supporting this article is included in the manuscript. For further information, you may contact the corresponding author.
